# Investigation of attachment and proliferation of MG-63 osteoblast-like cells to titanium disks in the presence of iPRF (injectable platelet-rich fibrin) or CGF (Concentrated Growth Factor): A laboratory study

**DOI:** 10.34172/japid.026.3383

**Published:** 2025-12-13

**Authors:** Maryam Torshabi, Zeinab Rezaei Esfahrood, Behzad Houshmand, Seyed Hosein Tabatabaee

**Affiliations:** ^1^Department of Dental Biomaterials, School of Dentistry, Shahid Beheshti University of Medical Sciences, Tehran, Iran; ^2^Department of Periodontics, School of Dentistry, Shahid Beheshti University of Medical Sciences, Tehran, Iran

**Keywords:** Cell adhesion, Cell proliferation, Dental implants, Osteoblasts, Titanium

## Abstract

**Background.:**

The attachment of the dental implant to the host bone (osteointegration) is considered a critical factor in the success of implant treatment. Osteoblasts are the primary cells in the osteointegration process. Today, compounds containing growth factors are used to shorten osteointegration time and increase the treatment success rate. This study evaluated the effects of iPRF (injectable platelet-rich fibrin) and CGF (concentrated growth factor) platelet extracts on the attachment and proliferation of MG-63 osteoblast-like cells over titanium disk surfaces.

**Methods.:**

Titanium pieces with a length of 12.1 mm and a width of 3.8 mm were prepared. The MG-63 cell viability, attachment, and proliferation on titanium disks were evaluated in the presence of prepared extracts from iPRF and CGF using methyl thiazolyl tetrazolium (MTT) assay and scanning electron microscopy (SEM).

**Results.:**

The results of the direct exposure of different concentrations of extracts on MG-63 cells cultured on the polystyrene surface of cell culture plates in the absence of fetal bovine serum (FBS) showed no significant difference between different concentrations of iPRF and CGF extracts in the first 24 hours after exposure (*P*>0.05). However, 48 hours after exposure, CGF extracts showed better effects (*P*<0.05). In the first 24 hours, MG-63 cell attachment to the titanium disks was significantly higher after exposure to CGF compared to iPRF and the control group (*P*<0.05). Nevertheless, 48 hours after culturing, no significant differences were observed in MG-63 survival, proliferation, and attachment between iPRF, CGF, and the control group (absence of iPRF and CGF) (*P*>0.05).

**Conclusion.:**

The results showed a better short-term (first 24 hours after exposure) effect of CGF on primary cell survival, attachment, and proliferation compared to iPRF; however, this superiority disappeared over time.

## Introduction

 Titanium implants are the preferred treatment for full and partial edentulous reconstruction.^1^ Attaching the titanium implant to the host bone is considered a key factor in the success of the implant treatment.^2^ This direct attachment between the living bone and the surface of the implant on the electron microscope scale is called osteointegration.^3^ The primary interaction between the implant surface, extracellular matrix proteins, and osteoblasts has a decisive effect on the osseointegration process.^4^

 In recent years, the use of regenerative treatment methods in dentistry has become increasingly evident in improving the standards of periodontology and implant treatments.^5^ One regenerative method for lost tissue is the use of platelet concentrates, such as platelet-rich plasma (PRP) and platelet-rich fibrin (PRF), which contain concentrated growth factors (CGFs) from the patient’s blood.^6^ It has been reported that PRP facilitates angiogenesis, hemostasis, osteogenesis, and bone growth and has an antibacterial effect. The high concentration of growth factors in PRP accelerates tissue repair across many organs with minimal side effects.^7^ However, limitations in the regeneration ability of this concentrate have been demonstrated because, in its preparation process, anticoagulants are added, known as tissue regeneration suppressors.^8,9^ The fibrinolytic system can result in the recall of mesangial stem cells and prevent proteolysis, thus improving the healing process.^7^

 Another regenerative treatment method is the application of injectable platelet-rich fibrin (iPRF). iPRF is a liquid form of PRF that coagulates after preparation.^10^ The advantage of iPRF over PRP can be attributed to the speed and time of centrifugation, which is reduced, and centrifuge tubes contain more hydrophobic material to reduce the coagulation time.^11^ iPRF is a mixture of concentrated platelets and leukocytes that can induce the regeneration of both soft and hard tissues.^12^ Advantages of PRF over PRP include shorter preparation time and no need for anticoagulants. In addition, unlike PRF, in PRP, growth factors are not trapped in a fibrin network, leading to the rapid release of growth factors.^13^ Also, PRP, unlike PRF, does not contain leukocytes, which makes it unable to reproduce growth factors after initial release.^13^

 Another form of platelet product is CGF, an autologous L-PRF obtained by centrifuging blood samples in vacuum tubes with a special centrifuge similar to the PRF centrifuge.^14^ Both the iPRF and CGF contain significant amounts of growth factors compared to PRP and have a greater ability to induce angiogenesis and thus increase wound regeneration.^15^ PRP, PRF, and CGF are widely used today to reduce the time between the bone graft and implant placement and to increase the treatment success rate.^16^ What surface features are involved in different cellular responses, and what effect they have on the results of the comparative laboratory investigation of the osteogenic properties of titanium implants, is still a challenge.^17^

 The hypothetical mechanisms for the superiority of CGF over iPRF include the presence of a large, concentrated, and growth factor-rich fibrin matrix in CGF and low levels of fibrinogen, thrombin, and factor XIII that increase fibrin clot cohesion and, at the same time, increase its tensile strength and stability.^18^ On the other hand, the physiological concentration of growth factors, in contrast to supraphysiological concentrations of growth factors in CGF, simplicity of preparation, and less variability in the quality of PRF have been hypothesized for the superiority of iPRF over CGF.^19^ To the best of our knowledge, no study has yet compared the effects of CGF and iPRF on the behavior of bone cells on the surface of titanium implants. This study aims to compare the effects of these platelet extracts on the attachment and proliferation of MG-63 osteoblast-like cells in a laboratory environment.

## Methods

###  Cell culture 

 MG-63 cells (C555; Pasteur Institute of Iran, Tehran, Iran) were cultured in 25-mL cell culture flasks (SPL Life Sciences, Pocheon, Korea) in Dulbecco’s Modified Eagle Medium (DMEM) containing 10% fetal bovine serum (FBS) and 1% antibiotic (penicillin-streptomycin) (Bioidea, Tehran, Iran). The cells were kept at 37 °C in an incubator with 5% CO_2_ and 95% humidity.

###  Preparation of iPRF and CGF

 The samples were obtained from healthy male volunteers aged 18‒25 years. Venous blood (9 mL) was collected in iPRF tubes (SPL Life Sciences, Pocheon, Korea). The Ghanaati protocol was used to prepare the iPRF, which was obtained by centrifugation (Rotina 380R; Hettich, Tuttlingen, Germany) at 700 rpm for three minutes, without adding any anticoagulant. The orange iPRF concentrate was immediately aspirated into a syringe.

 The Sacoo protocol was used to prepare CGF, which was obtained by centrifugation at 700 rpm for 12 minutes without adding any anticoagulant.

 Immediately after preparing the samples, in sterile conditions, 1 mL of iPRF and CGF samples was transferred to 6-well cell culture plates (SPL Life Sciences, Pocheon, Korea) and mixed with DMEM (5 mL). The corresponding plate was kept in an incubator with 5% CO_2_ and 95% humidity at 37 °C. The supernatant (extract) was collected 72 hours after incubation and kept at -70 °C until further experiments.

###  Preparation and processing of titanium disks

 Five commercial-grade titanium disks measuring 12.1 mm in length and 3.8 mm in width (KFP Dental Co., Tehran, Iran) were prepared. In each piece, 7.3 mm of the length was prepared as a cylinder, and the rest as flat. The parts were mechanically polished, and the flat part was used as a titanium disk. These disks were placed in the plate after being sterilized in an autoclave.

###  MG-63 viability and proliferation evaluation using MTT assay

 MTT colorimetric test was used to investigate and compare the MG-63 osteoblast-like cell viability, proliferation, and cytotoxicity at different concentrations of iPRF and CGF. On the first day of the study, the studied cells, which were in the logarithmic phase of growth, were carefully counted and cultured (2500 cells in 100 µL per well) in a 96-well culture plate. The plates were then incubated for 24 hours. On the second day, concentrations of 25, 50, and 100% of iPRF and CGF extracts (diluted with DMEM) with or without 10% FBS were added to the cells separately. Cells without iPRF or CGF treatment were considered negative controls.

 Viability, proliferation, and cytotoxicity were assessed at 24 and 48 hours after exposure. The culture medium in each well was replaced with DMEM containing 10% MTT dye (Sigma-Aldrich, Darmstadt, Germany). MTT dye was drained from each well after formazan crystals formed, and the same volume of DMSO (Sigma-Aldrich, Darmstadt, Germany) was added to each well. Then, an ELISA reader measured the absorbance of the colored solutions (Anthos 2020, Vienna, Austria). Cell viability was determined by dividing the average optical absorbance of the experimental groups by that of the control. The 70% viability was considered cytotoxic according to ISO-10993-5.

###  MG-63 attachment and proliferation evaluation using MTT assay 

 Sterile disks were placed in 24-well culture plates without cell-adhesive coating. Approximately 200,000 pre-prepared MG-63 cells were directly cultured on titanium disks in the presence of 100% concentration of iPRF or CGF (without the presence of FBS). Cells cultured on disks without iPRF or CGF were considered controls.

 At the time of examination (24 or 48 hours after incubation), the viability and proliferation were measured using the MTT assay as mentioned earlier.

###  Evaluation of MG-63 attachment to a titanium disk using scanning electron microscopy (SEM)

 For SEM sample preparation, titanium disks (cultured with MG-63 cells and treated with iPRF or CGF for 24 hours) were washed once with phosphate-buffered saline (PBS) (Bioidea, Tehran, Iran) and then fixed with 2.5% glutaraldehyde (Sigma-Aldrich, Darmstadt, Germany) and kept overnight in the refrigerator. After being washed with PBS, the samples were dehydrated with different concentrations of alcohol (30%, 40%, 50%, 60%, 70%, 80%, 90%, and 100%). After air-drying and gold-coating, the morphology and cell attachment to the titanium disks were evaluated under an electron microscope (AIS-2300CSEM, Seron Technology, Gyeongsu-daero, Korea).

###  Statistical analysis

 The GraphPad Prism software version 9 (GraphPad Software, Inc., La Jolla, USA) was used for the analysis. Comparisons were performed using the one-way analysis of variance (ANOVA) with post hoc Tukey-Kramer analysis. The level of statistical significance was set at 0.05 (*P* < 0.05).

## Results

###  Effect of iPRF or CGF extract on MG-63 viability and proliferation

**Figure 1:**
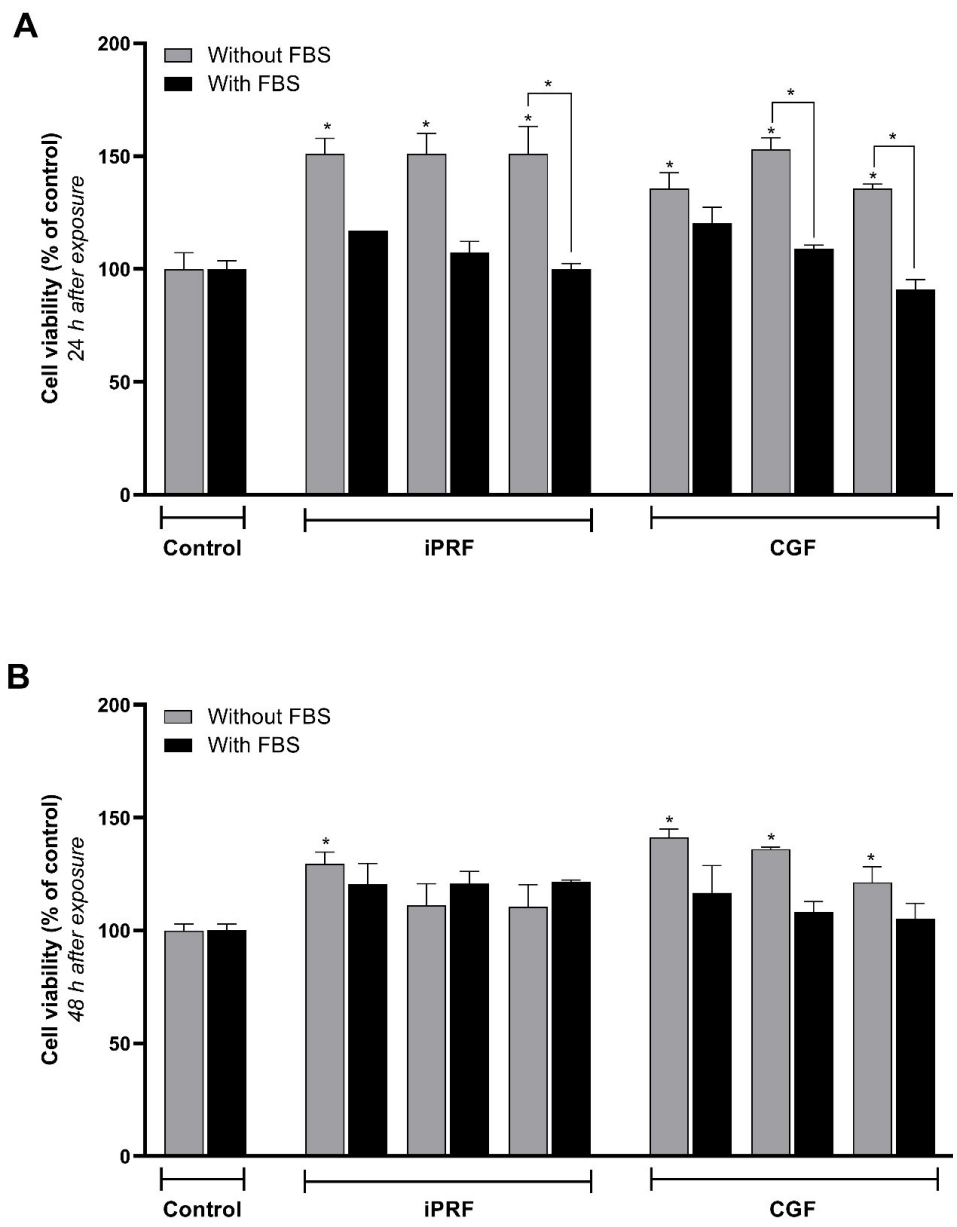
The effect of different concentrations of iPRF and CGF on the viability and proliferation of MG-63 cells at 24 h (A) and 48 h (B) after exposure. The stars on the columns indicate the significance of the difference between the target group and the control group (100% viability), and the stars between the two columns indicate the significance of the difference between the two target groups (cell proliferation) (*P* < 0.05)

Figure 1A presents a comparison of the effects of iPRF and CGF extracts on MG-63 viability and proliferation 24 hours after exposure. There was no significant difference in viability percentages between the control group (without CGF or iPRF, 100% viability) and the iPRF and CGF groups in the presence of FBS (*P* = 0.998). Both iPRF and CGF significantly increased cell viability (by 50%) in the absence of FBS compared to the control group (*P* < 0.001). However, the effects of iPRF and CGF were not significantly different (*P* = 0.999).

 No significant differences were found between the concentrations of 25 (*P* = 0.998), 50 (*P* = 0.999), and 100% (*P* = 0.454) iPRF and CGF after 24 hours of exposure. There were no significant differences between CGF and iPRF in terms of the effect on cell viability and proliferation (*P* = 0.999). It also seemed that iPRF and CGF without FBS had more favorable effects on viability and proliferation. iPRF at 25% and CGF at 25% and 50% concentrations significantly increased cell viability and proliferation. This effect was more pronounced in the absence of FBS than in its presence (*P* = 0.999).

 As seen in Figure 1B, 48 hours after exposure of the studied materials to MG-63 cells, there was a significant difference in the percentage of viability between the control group (without CGF and iPRF, 100% viability) and the iPRF and CGF groups in the presence of FBS (*P* = 0.031 and *P* = 0.001, respectively) and in the absence of FBS. There was no significant difference in viability between the control group and iPRF and CGF at 25% (*P* = 0.278 and *P* = 0.999, respectively), 50% (*P* = 0.340 and *P* = 0.998, respectively), and 100% (*P* = 0.354 and *P* = 0.667, respectively) concentrations in the presence of FBS. According to the 48-hour exposure results, there was no difference between the 25%, 50%, and 100% concentrations of CGF and the 100% concentration of iPRF. It also seemed that the three concentrations of CGF and the 100% concentration of iPRF in the absence of FBS had better effects on the viability and proliferation of MG-63 cells.

###  Effect of iPRF or CGF extract on the attachment and proliferation of MG-63 cells on titanium disks

**Figure 2:**
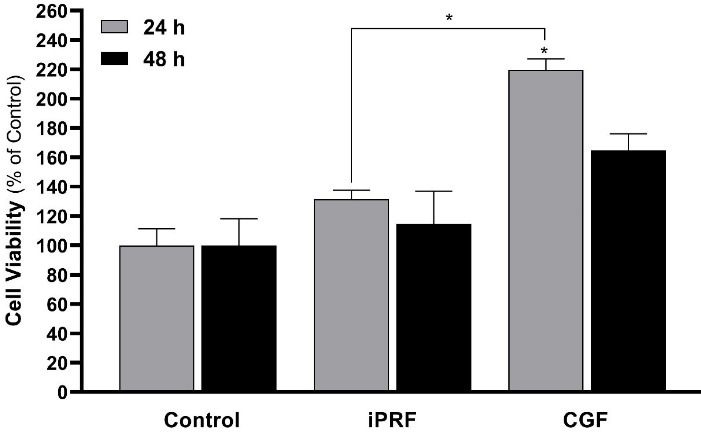
The effect of a 100% concentration of iPRF and CGF on attachment, viability, and proliferation of MG-63 cells at 24 h and 48 h after implantation on disks. The stars on the columns show the significance of the difference between the target group and the control group (100% survival), and the stars between the two columns show the significance of the difference between the two target groups (*P* < 0.05)

Figure 2 presents a comparison of the attachment of MG-63 cells to the titanium disk after 24 hours of exposure to iPRF and CGF. There was a significant improvement in exposure to CGF compared with the control group only 24 hours after exposure (*P* = 0.002).

 In general, the results showed that the effect of CGF on attachment, survival, and primary cell proliferation was greater than that of iPRF and control in the first 24 hours of implantation and at the cell‒disk interface, but this superiority diminished over time.

###  Effect of iPRF or CGF extract on MG-63 cell attachment and proliferation 

**Figure 3:**
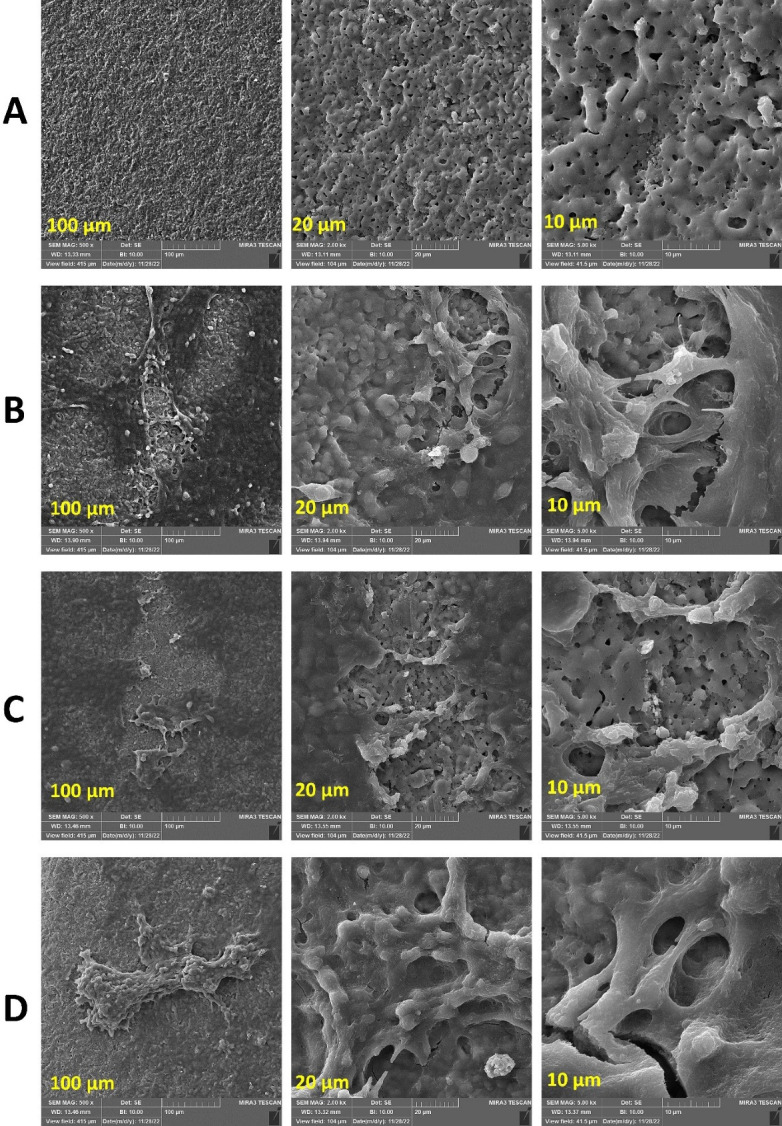
Group A: implant disk without cells. Group B: implant disks with MG-63 cells without iPRF and CGF. Group C: implant disk with MG-63 cells in the presence of iPRF. Group D: implant disk with MG-63 cells in the presence of CGF

 Microscopic studies (Figure 3) showed that the cells exhibited better attachment in the presence of CGF compared to other groups. SEM images of the surface of a titanium disk without cells (Figure 3A) showed irregular hollows with a fractal structure and random topography of the surface. Figure 3B shows the titanium disk with adherent MG-63 cells without iPRF and CGF, with the cells having sound and normal morphology and spreading irregularly over the disk surface.

 In the presence of iPRF and CGF (Figures 3C and 3D ), more adherent cells with normal morphology and better attachment are observed, presenting a broader and denser distribution compared to the untreated disk. All the groups presented the typical shape of MG-63 cells. However, in the presence of CGF, more attachment and cellular extensions were observed, along with a higher rate of cellular growth and proliferation.

## Discussion

 The present study indicated that both iPRF and CGF increased MG-63 cell viability compared to the control group in the absence of FBS. However, no significant difference in MG-63 cell viability was observed between iPRF and CGF concentrations with FBS compared to the control group after 24 hours. The present study also showed no significant differences in MG-63 cell viability between iPRF and CGF extracts at different concentrations and the FBS and control groups. The observed improvement in MG-63 cell viability in both iPRF and CGF extracts compared to the control group was consistent with a previous study that reported compounds containing PRP, PRF, and CGF could reduce the time between bone graft placement and implant placement and thus increase the treatment success rate.^16^ In a study that investigated the effect of PRP, PRF, and CGF on the healing of the parietal bone of rabbits, bone density and volume in the sixth and twelfth weeks were higher in the experimental groups compared to the control group, consistent with our study. Still, no significant difference was observed between the experimental groups.^16^ A total of 23 studies were reviewed systematically for PRF^20^ and CGF.^21^ According to some studies, platelet products have a positive effect on the primary stability of the implant.^22-24^ In most studies, the positive effects of these treatments were mentioned, but the researchers suggested that more studies are needed to substantiate this evidence.

 The mechanisms underlying iPRF’s effect on MG-63 cell viability include the release of various growth factors and its ability to cover the implant surface before clotting due to its liquidity. Furthermore, iPRF forms a stable fibrin layer on the implant surface, which contains platelets and leukocytes.^25^ This causes a kind of biological layer to be created on the surface of the implant, which makes the neutral surface of the implant biologically active. The mechanisms of the effect of CGF on MG-63 cell viability include its ability to trap a large number of cells, including stem cells that are responsible for the continuous production and release of soluble mediators in CGF (including growth factors and matrix metalloproteinases).^26^

 The findings of the present study indicated a significant difference in MG-63 cell viability between 100% iPRF and the control group after 48 hours. In contrast, a dose‒response relationship was observed between different concentrations of CGF and the control group at the same time point. These findings indicated that MG-63 cell viability increased with increasing concentrations of the CGF extract. However, CGF extract induced a significantly higher MG-63 viability only after 24 hours, and the effects of both the iPRF and CGF were not comparable after 48 hours. Studies reporting the clinical use of CGF are not abundant. Among studies that investigated the interaction between CGF and implants is the research by Faramarzi et al.^27^ The 40% and 80% concentrations of CGF improved the survival of human gingival fibroblasts (HGFs). Similarly, in the present study, the CGF group was significantly superior to the control and the iPRF groups in the presence of titanium disks. A study investigated the effects of PRP and PRF on the adhesion of osteoblasts pretreated with bisphosphonates on titanium implant surfaces.^28^ The positive effects of these two substances were also observed in patients consuming bisphosphonate, in a way that they reduced the adverse effects of zoledronic acid on the adhesion of osteoblasts. However, in the present study, the effect of iPRF at 24 and 48 hours was almost similar to that of the control group. In a pre-test stage, they also examined differences in the effectiveness of various PRF concentrations, concluding that the 2.5% PRP and 5% PRF concentrations had a better effect than others. However, in the pre-test stage of the present study (i.e., in the absence of titanium disks), no significant difference was observed between various CGF and iPRF concentrations, which may be due to the different concentrations used in the two studies.

 The present study indicated that CGF resulted in better MG-63 cell attachment and proliferation on the titanium disk compared to iPRF. In some studies, CGF has had a positive effect on implant stability and osseointegration,^29^ whereas in others, CGF has not been significantly different from the control group.^30^ Regarding cell attachment, another study similar to the present study confirmed that CGF significantly improved the adhesion of endothelial cells on implants.^26^ Similar to the findings of the present study, a study examined the response of MG-63 cells adjacent to iPRF on titanium disks. Cell proliferation, alkaline phosphatase production, and mineralization from day 1 to day 21 in both experimental and control groups were significantly higher than in the control group. At the same time, in the present study, iPRF did not have a significant effect on titanium disks.^31^ In a study, the authors placed implants in dogs’ femoral bone defects.^32^ CGF and PRF bone were grafted to the bone defect area. Compared with PRF, CGF showed a higher and better new bone formation rate in peri-implant bone defects, consistent with the present study. Li et al^33^ applied PRF and CGF in the tooth extraction sockets of rabbits. Overall, CGF was more effective than PRF at promoting extraction socket healing. They stated that the reason may be that the rich fibrin fibers and fibrinogen in CGF play an essential role in promoting the healing of tooth extraction sockets. Moreover, CGF significantly outperformed PRF in promoting osteogenesis at later stages.

 CGF is known to have higher tensile strength, growth factor concentration, and viscosity than PRF,^13^ which can explain its better performance in the present study. Its higher tensile strength and viscosity result in better protection of growth factors against proteolysis.^16^ A previous study showed that iPRF works better compared to PRP in cell migration and growth factor expression.^34^ The authors cited that this effect was related to the difference in the preparation protocols of PRP and iPRF.^34^ In the present study, CGF and iPRF were prepared using two separate protocols, which may justify the difference that exists in the performance of CGF and iPRF. CGF has been reported to contain more growth factors than PRF and to have a more rigid fibrinogen structure.^32^ Also in SEM analysis, CGF shows a thicker and more regular pattern of fibrin than PRF and has a thicker and denser arrangement of fibrinogen fibers per unit area than PRF.^32^ Based on SEM examination, CGF gels also contain thicker fibrin fibers than PRF gels.^35^ Moreover, CGF contains approximately 1.5 times more vascular endothelial growth factor (VEGF^)^ than PRF.^32^ The growth factors in the dense structure of CGF fiber have the characteristic of slower release.^36^ Compared with PRF, CGF not only has a higher fibrinogen but also has a more stable fibrinogen network, which can prevent plasma-mediated degradation.^32^ CGF shows greater benefits than PRF in osteogenesis, resulting in efficient bone induction and tissue regeneration in its presence.^33^ In maxillary sinus augmentation and alveolar bone grafts, CGF is more effective in the regeneration of blood vessels than PRF.^37^

 In contrast to the findings of this study, it was previously shown that coating the titanium surface with iPRF provides a biological advantage at the cellular level by increasing cell activity and proliferation, which may lead to greater bone-to-implant contact and faster, more robust osseointegration.^31^ One of the reasons for the effectiveness of iPRF is its strong antibacterial activity.^38^ Although the microbial test was not included in the current study, it seems that the role of factors such as iPRF goes beyond accelerating cell proliferation and activities, and their antimicrobial properties may be one of the factors affecting their performance.^31,38^

 One limitation of the present study is the lack of a clinical phase, which could be considered in future plans. To our knowledge, this study is the first to compare the effects of iPRF and CGF on the attachment and proliferation of osteoblast-like cells on titanium disks in vitro. We concluded that CGF has a greater impact on cell attachment and early proliferation than iPRF and is likely a favorable option in cases where immediate implant loading is required.

## Conclusion

 The findings of the present study showed that both the iPRF and CGF extracts were effective in improving MG-63 cell viability, proliferation, and attachment to the titanium disk. However, the maximum effect of CGF on viability was achieved more quickly than with iPRF. Better overall performance was observed when different percentages of iPRF and CGF were used in the absence of FBS than in its presence.

## Competing Interests

 The authors declare that they have no competing interests.

## Data Availability

 The datasets used and analyzed during the current study are available from the corresponding author on reasonable request.

## Ethical Approval

 Written informed consent was obtained from the participants. The study was conducted in accordance with the institutional codes of ethics and the Declaration of Helsinki. This study was approved by the Ethics Committee of the Shahid Beheshti University of Medical Sciences, School of Dentistry (code: IR.SBMU.DRC.REC.1400.167).
